# Intravital imaging of Ca^2+^ signals in lymphocytes of Ca^2+^ biosensor transgenic mice: indication of autoimmune diseases before the pathological onset

**DOI:** 10.1038/srep18738

**Published:** 2016-01-06

**Authors:** Soichiro Yoshikawa, Takako Usami, Junichi Kikuta, Masaru Ishii, Tetsuo Sasano, Koji Sugiyama, Tetsushi Furukawa, Eiji Nakasho, Hiroshi Takayanagi, Thomas F. Tedder, Hajime Karasuyama, Atsushi Miyawaki, Takahiro Adachi

**Affiliations:** 1 Department of Immune Regulation, Tokyo Medical and Dental University, Tokyo, 113-8519, Japan; 2 Laboratory of recombinant animals, Medical Research Institute, Tokyo Medical and Dental University, Tokyo, 101-0062, Japan; 3 Immunology and Cell Biology, Graduate School of Medicine, Osaka University, Suita 565-0871, Japan; 4 Department of Biofunctional Informatics, Tokyo Medical and Dental University, Tokyo 113-8519, Japan; 5 Department of Bio-informational Pharmacology, Medical Research Institute, Tokyo Medical and Dental University, Tokyo 113-8510, Japan; 6 R&D Group, Scientific Solutions Product Development Division, Olympus Corporation, Hachioji-shi, Tokyo 192-8507, Japan; 7 Department of Immunology, Graduate School of Medicine and Faculty of Medicine, The University of Tokyo, Tokyo 113-0033, Japan; 8 Department of Immunology, Duke University Medical Center, Durham, NC 27710; 9 Laboratory for Cell Function and Dynamics, Advanced Technology Development Group, Brain Science Institute, RIKEN, Wako, Saitama 351-0198, Japan; 10 Department of Immunology, Medical Research Institute, Tokyo Medical and Dental University, Tokyo, 113-8510, Japan

## Abstract

Calcium ion (Ca^2+^) signaling is a typical phenomenon mediated through immune receptors, such as the B-cell antigen receptor (BCR), and it is important for their biological activities. To analyze the signaling of immune receptors together with their *in vivo* dynamics, we generated stable transgenic mice with the Föster/fluorescence resonance energy transfer (FRET)-based Ca^2+^ indicator yellow cameleon 3.60 (YC3.60), based on the Cre/loxP system (YC3.60^flox^). We successfully obtained mice with specific YC3.60 expression in immune or nerve cells as well as mice with ubiquitous expression of this indicator. We established five-dimensional (5D) (x, y, z, time, and Ca^2+^) intravital imaging of lymphoid tissues, including the bone marrow. Furthermore, in autoimmune-prone models, the CD22^−/−^ and C57BL/6- lymphoproliferation (lpr)/lpr mouse, Ca^2+^ fluxes were augmented, although they did not induce autoimmune disease. Intravital imaging of Ca^2+^ signals in lymphocytes may improve assessment of the risk of autoimmune diseases in model animals.

Calcium ions (Ca^2+^) are universal second messengers with multiple functions in most cells. In the immune system, stimulation of immune receptors, such as the B-cell antigen receptor (BCR), induces intracellular Ca^2+^ mobilization concomitant with other signaling events, such as phosphorylation of cellular substrates^1^,^2^,^3^,^4^,^5^. Ca^2+^ signaling is involved in regulating the mitogen-activated protein kinase nuclear factor of activated T cells, and nuclear factor-κB pathways in B cells, and it is crucial for B-cell development and function during humoral immune responses^1^,^3^.

To date, synthetic calcium indicators, such as Fluo-4, are being used to analyze immune receptor-mediated Ca^2+^ signaling. Although these synthetic compounds exhibit high resolution, their use is toxic and their intracellular retention is limited. To solve these problems, genetically encoded Ca^2+^ indicators, such as GCaMP3 and Yellow Cameleon 3.60 (YC3.60), have been generated^6^,^7^. These indicators are suitable for long-term, repeated measurements and are used for neuronal imaging *in vivo*^8^,^9^,^10^. The GCaMP3 is a Ca^2+^ indicator based on a single circularly permuted green fluorescent protein (GFP)^7^. As the readouts of GCaMP3 directly depend on fluorescence intensity, this indicator is suitable for use in stationary cells. However, calibrating the baseline for migrating cells, such as immune cells, is very difficult. On the other hand, YC3.60 is a double-chromophore indicator that employs Förster/fluorescence resonance energy transfer (FRET) between cyan fluorescent protein (CFP) and a circularly permuted variant of the yellow fluorescent protein (YFP) Venus^6^. Ca^2+^ signaling can be monitored by measuring the ratio of YFP to CFP (YFP/CFP). FRET-based ratiometric indicators, including YC3.60, can be corrected for unequal sensor expression. Thus, ratiometric sensors, such as YC3.60, are suitable for *in vivo*, whole-body imaging in mice, particularly for migrating immune cells.

Genetically-encoded, fluorescent proteins have been employed for *in vivo* study of immune cells. Visualization of T and/or B cells in lymphoid tissues has revealed details of their functions under physiological conditions^11^,^12^,^13^,^14^,^15^. During activation, most immune cells migrate to certain tissues and encounter various cells at different developmental stages; in these tissues, they receive and/or emit signals via soluble factors or cellular interactions to further modulate their functions. Therefore, to understand the mechanisms of the complex immune system, it is necessary to not only dissect the interactions but also to analyze the signaling mediated by immune cells. Although transgenic mice expressing the FRET-based Ca^2+^ indicator TN-XLL, under the control of the ubiquitously active hybrid CMV enhancer/chicken β-actin (CAG) promoter, have been generated, the immune cells in these mice have not expressed TN-XLL^16^. To solve this problem, retrovirally transduced and improved FRET-based Ca^2+^ indicators were used for intravital analysis of T cells^17^. However, a stable transgenic mouse line expressing a FRET-based Ca^2+^ biosensor has not yet been generated. Thus, the extent of visualization of cellular signaling in immune cells *in vivo* remains limited.

Previously, we employed YC3.60 to create a system to detect Ca^2+^ mobilization within the immune system^18^,^19^ and demonstrated that Ca^2+^ mobilization in B-cell lines could be strongly detected. Recently, we further developed this system and established a transgenic mouse line that conditionally expressed YC3.60 to visualize the temporal and spatial dynamics of Ca^2+^ signaling within immune cells. This transgenic mouse line allowed us to analyze specific cell functions under both normal physiological and pathological conditions.

## Results

### Generation and characterization of conditional YC3.60 expression mice

**Figure 1:**
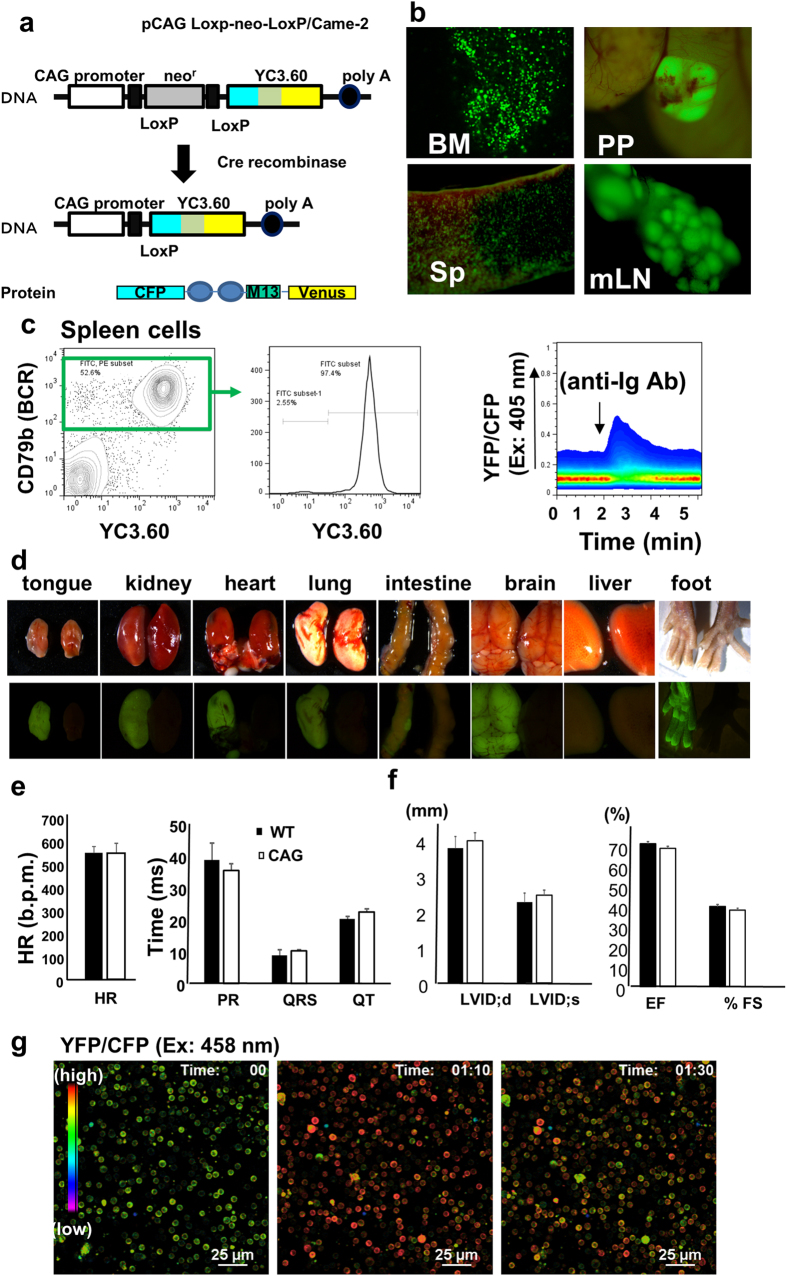
Characterization of YC3.60^flox^/CD19-Cre and YC3.60^flox^/CAG-Cre mice. (**a**) Schematic diagram of the conditional YC3.60 expression construct. (**b**) Representative images of YC3.60^flox^/CD19-Cre mouse lymphoid tissue. Peyer’s patches (PP), bone marrow (BM), mesenteric lymph node (mLN), and spleen (Sp) were analyzed by fluorescent microscopy (n = 3 mice). YC3.60-expressing cells are shown in green. (**c**) BCR-mediated Ca^2+^ mobilization in splenic B cells. Splenic cells were prepared from YC3.60^flox^/CD19-Cre mice. Percentages in Igβ^+^ cells are shown (middle panel). Ca^2+^ mobilization was determined by flow cytometry based on YC3.60 FRET. An anti-Igβ monoclonal antibody (mAb) (HM79) was added to the splenic cells at the indicated time point. Results are representative of at least five independent experiments (*n* > 5 mice). (**d**) YC3.60 expression in organs of CAG-Cre/YC3.60 mice. Bright-field and fluorescent images of isolated organs from CAG-Cre/YC3.60 (left) mice and wild-type (right) mice are shown. (**e**) ECG analysis of ubiquitous YC3.60 expression mice. Heart rates (left) and ECG parameters (right) are shown. (**f** ) UCG analysis of CAG-Cre/YC3.60^flox^ expression mice. UCG parameters are shown. (e and f) CAG-Cre/YC3.60 (*n* = 3 mice) and wild-type (*n* = 5 mice) are indicated by open and hatched bars, respectively. Mean and SD are shown. No significant differences were noted in any parameters (P > 0.05). (**g**) Ionomycin-induced Ca^2+^ fluxes in splenic cells from the ubiquitous YC3.60 expression mouse. Ionomycin (final concentration 5 μmol/L) was added in splenic cell culture 50 s after starting observation. Representative ratiometric images (YFP/CFP at excitation of 458 nm) are shown that were obtained using confocal microscopy. Scale bar, 25 μm. Frame = 64.

We tried to generate transgenic mice with the YC3.60 gene (Supplementary Fig. S1a) under control of a CAG enhancer/promoter that initiates ubiquitous expression of the gene. However, we failed to do so, despite several trials. Therefore, we tried to generate conditional YC3.60 transgenic mice based on the Cre/loxP system (YC3.60^flox^ mice; Fig. 1a). The YC3.60 gene is not expressed in these mice because a neomycin phosphate transferase gene is inserted between the CAG enhancer/promoter^20^ and YC3.60 gene. After crossing with CD19-Cre mice in which Cre recombinase is expressed under the regulation of the CD19 gene^21^, the YC3.60 gene was specifically expressed in B cells while considering CD19 as a typical B-cell marker. We obtained two mouse lines that expressed YC3.60 in B cells (YC3.60^flox^/CD19-Cre mice), although one of these (line No.1) expressed YC3.60 in only 3% of splenic B cells (Supplementary Fig. S1b). We further analyzed another YC3.60^flox^ line (line No. 2) because it expressed YC3.60 in most B cells upon crossing with the CD19-Cre line (Supplementary Fig. S1b).

The YC3.60-expressing B cells were detected in tissues that were abundant in B cells, such as the spleen, lymph nodes, bone marrow, and Peyer’s patches (Fig. 1b). The fluorescence intensity of YC3.60 was high enough to detect YC3.60-expressing cells even in tissues with high levels of autofluorescence, such as the intestine. The B220^+^ B-cell population in the spleen of YC3.60^flox^/CD19-Cre mice was almost the same as that observed in wild-type mice (Supplementary Fig. S1c). YC3.60 expression in germinal center (GC) B cells (GL-7^+^) and plasmablasts/plasma cells (CD138^+^) in the spleen is slightly higher than that of naïve B cells (Supplementary Fig. S1d). Spleen weight and total-cell, T-cell, and B-cell numbers in the spleen of YC3.60^flox^/CD19-Cre mice were consistent with those of wild-type mice. (Supplementary Fig. S1e and f). In addition, we confirmed that serum immunoglobulin M (IgM) and immunoglobulin G (IgG) levels in YC3.60^flox^/CD19-Cre mice were consistent with those in wild-type mice (Supplementary Fig. S1g). Upon immunization with alum-absorbed ovalbumin (OVA) (OVA/alum), serum anti-OVA IgG in YC3.60^flox^/CD19-Cre mice was normally induced. These results suggest that development of B cells and their immune responses in YC3.60^flox^/CD19-Cre mice are almost the same as those in wild-type mice.

Furthermore, we identified the integration site of the YC3.60 gene using inverse polymerase chain reaction (PCR). The YC3.60 construct was inserted into chromosome 10 between the *Spock2* and *Chst3* loci at a 20-kb distance from each gene (Supplementary Fig. S1h). The insertion into chromosome 10 ensured that the integrated YC3.60 construct did not interrupt the coding regions of other genes. Furthermore, the YC3.60 expression has been perfectly matched with this locus in this transgenic mouse line over eight generations, strongly suggesting that the YC3.60 gene was not inserted in any other locus.

We further analyzed YC3.60^flox^/CD19-Cre mice to validate the YC3.60 gene as a Ca^2+^ biosensor in immune cells. We assessed primary B cells from YC3.60^flox^/CD19-Cre mice to determine whether Ca^2+^ mobilization could be monitored by assessing YC3.60 FRET. We cultured spleen cells and evaluated BCR-induced Ca^2+^ mobilization. Upon BCR ligation, Ca^2+^ mobilization was detected in primary B cells (Fig. 1c), as previously shown in B-cell lines^18^. The detection of Ca^2+^ mobilization in primary B cells demonstrated that the YC3.60^flox^/CD19-Cre mice could be used to analyze B-cell signaling events.

To ensure tissue-specific expression, we further crossed the YC3.60^flox^ mouse line with the CD4-Cre line, which specifically expresses the *Cre* gene in T cells^22^. As expected, the YC3.60 gene is expressed in lymphoid tissues, such as the spleen, in the CD4-Cre/YC3.60^flox^ mouse (Supplementary Fig. S2a). To examine YC3.60 expression in other cells in addition to immune cells, we also crossed the YC3.60^flox^ line with a nestin-Cre mouse line in which Cre was specifically expressed in the central nervous system^23^. The YC3.60^flox^/nestin-Cre line specifically expressed YC3.60 in nerve tissues, such as the brain, eye, and tongue (Supplementary Fig. S2a and b). The nestin-cre/YC3.60^flox^ mice did not show YC3.60 expression in immune cells, such as T and B cells (Supplementary Fig. S2a). Thus, we successfully obtained conditional YC3.60 mice that expressed YC3.60 in immune and nerve cells along the specific Cre line.

To obtain mice that ubiquitously express YC3.60, we crossed the YC3.60^flox^ mice with the CAG-Cre mice^24^, in which the *Cre* gene is ubiquitously expressed under the control of the CAG enhancer/promoter. Consequently, YC3.60 is ubiquitously expressed in various immune and nerve cells of YC3.60^flox^/CAG-Cre mice (Fig. 1d). Because the first CAG-Cre/YC3.60^flox^ mouse was born on May 31, 2012, we observed normal breeding capability in ubiquitous YC3.60 mice, without any severe phenotypes over 2 years. However, FRET-based Ca^2+^ biosensor transgenic mice had previously revealed a minor heart disorder^16^. Therefore, we examined the hearts of the ubiquitous YC3.60 expression mice by electrocardiogram (ECG) and ultrasound echocardiogram (UCG). Ubiquitous YC3.60-expression mice, including a homozygous genotype, were found to be normal (Fig. 1e,f). Furthermore, spleen cells from a CAG-Cre/YC3.60^flox^ mouse showed Ca^2+^ fluxes during ionomycin treatment (Fig. 1g, Supplementary Video S1), indicating that immune cells of the ubiquitous YC3.60-expression mice express a functional Ca^2+^ biosensor. Thus, we obtained the ubiquitous YC3.60 Ca^2+^ biosensor expression mice without significant congenital cardiac abnormality.

### Intravital imaging of Ca^2+^ signaling in the spleen, Peyer’s patches, and bone marrow of non-immunized YC3.60^flox^/CD19-Cre mice

**Figure 2:**
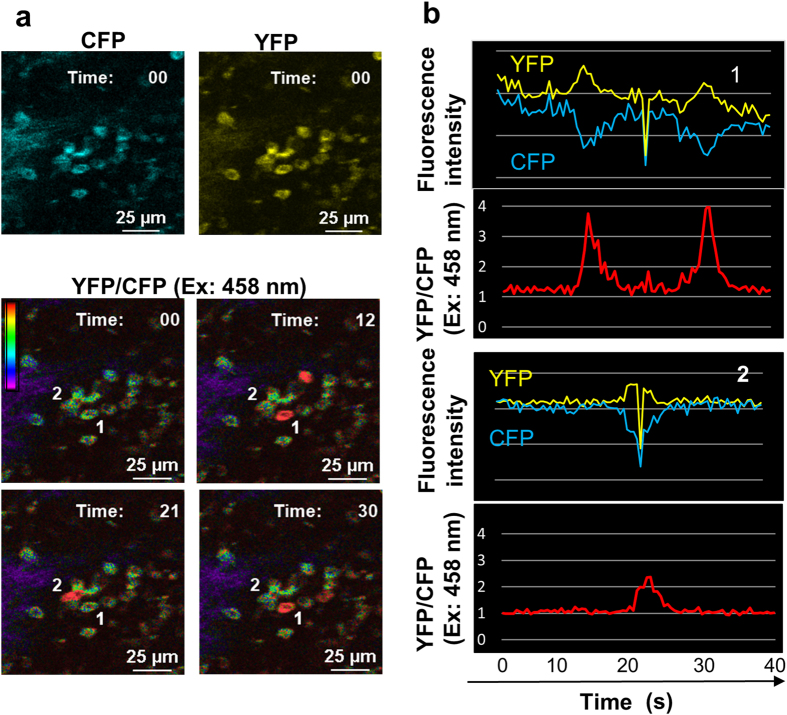
Intravital Ca^2+^ signaling images in the spleen of a YC3.60^flox^/CD19-Cre mouse. (**a**) Representative Ca^2+^ signaling images in the spleen of a YC3.60^flox^/CD19-Cre mouse are shown. A rainbow parameter indicates relative Ca^2+^ concentration. (**b**) Time courses for fluorescence intensities of CFP and YFP (Venus) and the YFP/CFP ratio at excitation of 458 nm in indicated cells in (**a**) are shown. Scale bar, 25 μm. Frame = 80.

To clarify B-cell signaling under physiological conditions, we attempted to establish intravital imaging of Ca^2+^ signaling in immune cells. We first used confocal laser microscopy to analyze the spleens of anesthetized YC3.60^flox^/CD19-Cre mice that had been maintained under specific pathogen-free (SPF) conditions without any immunization protocol. We observed fluorescence intensities of YFP and CFP with an excitation wavelength of 458 nm in splenic cells. A small number of B cells located at the periphery of the spleen exhibited temporal, inversely-correlated fluorescence intensities of YFP and CFP because of FRET, indicating transient increases in intracellular Ca^2+^ (Fig. 2a,b, Supplementary Video S2). Although fluorescence intensities of CFP and YFP were affected by the movement of the mice, these intensities were compensated for by ratiometric indications (Fig. 2b). Thus, we established a genetically encoded Ca^2+^ indicator mouse system to visualize Ca^2+^ signaling in a living animal.

**Figure 3:**
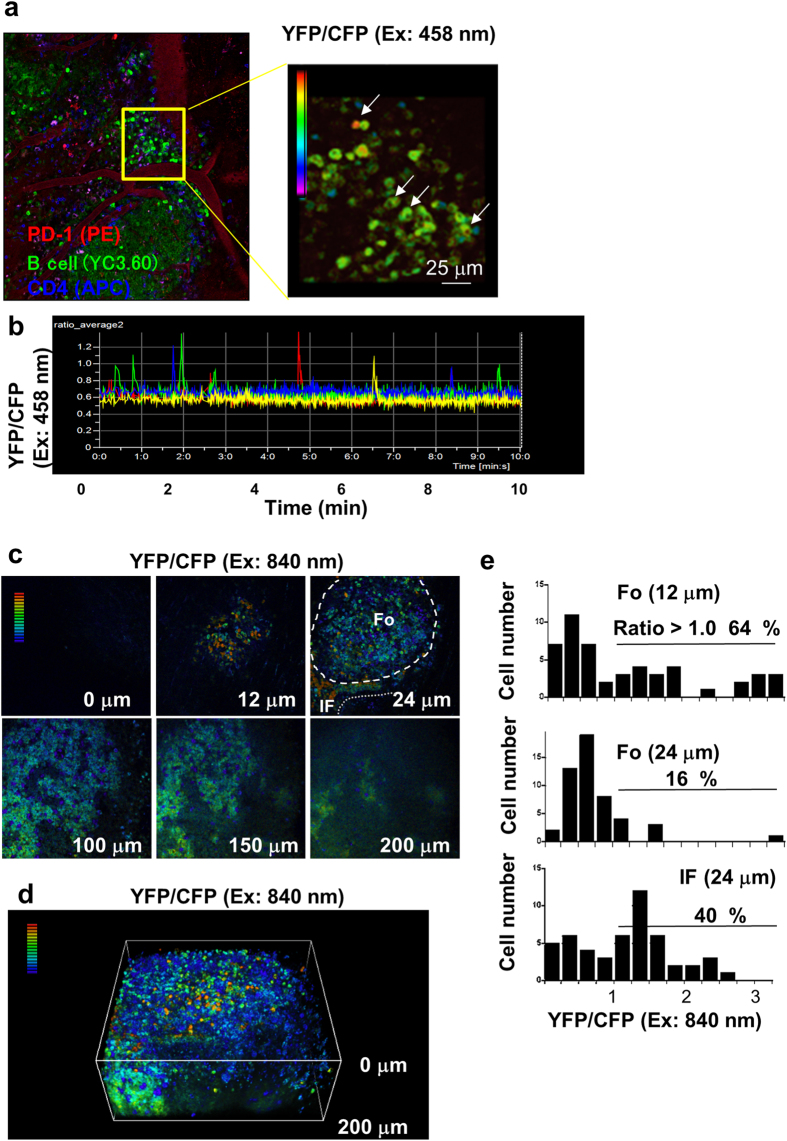
Representative Ca^2+^ signaling images in Peyer’s patches of a YC3.60^flox^/CD19-Cre mouse. (**a**) Image of Peyer’s patches of a YC3.60^flox^/CD19-Cre mouse after staining for CD4 and PD-1. YC3.60^+^ cells (green), CD4^+^ T cells (Blue), and PD-1^+^ cells (red) are shown (left). PE-conjugated anti-CD4 mAbs and Alexa647-conjugated anti-PD-1 mAs were intravenously injected 30 min before observation. Representative Ca^2+^ signaling image in Peyer’s patches of a YC3.60^flox^/CD19-Cre mouse (right). Only ratiometric images (YFP/CFP at excitation of 458 nm) are shown. Cells exhibiting FRET signals are indicated by arrows. Results are representative of at least three independent experiments (*n* = 3 mice). Scale bars, 25 μm. (**b**) Time courses of intracellular Ca^2+^ fluxes. Ratiometric intensities (YFP/CFP at excitation of 458 nm) of indicated cells in (**a**) were measured for 10 min with a no-delay program. Frame = 1129. (**c**) Z-stack analysis of intracellular Ca^2+^ concentrations of B cells in Peyer’s patches. Intravital imaging of Peyer’s patches was performed using two-photon microscopy. Ratiometric images (YFP/CFP at excitation of 840 nm) are shown. Z-stack images of 2-μm intervals up to a depth of 200 μm were obtained. Only representative images are shown. The follicle is indicated by a broken line. IF: interfollicular region. (**d**) Three-dimensional (3D) structure of B cells with intracellular Ca^2+^ concentrations in Peyer’s patches. 3D images based on Z-stack images (**c**) were obtained using Nis Elements software. (**e**) Distribution of intracellular Ca^2+^ concentrations of B cells of the indicated Z-stack images. *n* = 50.

Furthermore, we analyzed another secondary lymphoid organ, Peyer’s patches. Compared with the spleen, Peyer’s patches contain similar percentages of GC B cells and more plasmablasts/plasma cells (Supplementary Fig. S3a). Intravital imaging analysis of Peyer’s patches showed transient Ca^2+^ signaling in the interfollicular regions where T cells, including PD-1^+^ cells^25^, are abundant (Fig. 3a). Most of the Ca^2+^ signals in B cells were sporadic and short (Fig. 3b, Supplementary Video S3). To analyze the depth of the direction of Peyer’s patches under physiological conditions, we conducted intravital, two-photon excitation (2P) microscopic imaging, which allowed detection of Ca^2+^ signals in the cells located up to 200 μm from the surface of the Peyer’s patch. Structural analysis of Peyer’s patches by 2P microscopy allowed visualization of an entire follicle in the Peyer’s patch and showed the distribution of B cells with Ca^2+^ concentration. B cells having high intracellular Ca^2+^ concentrations were detected predominantly in the peripheral regions of the follicles and interfollicular regions (Fig. 3c–e, Supplementary Video S4). Small numbers of YC3.60^high^ cells appears to be GL-7^low^ cells and/or plasmablasts/plasma cells of CD138^+^ (Supplementary Fig. S3a).

**Figure 4:**
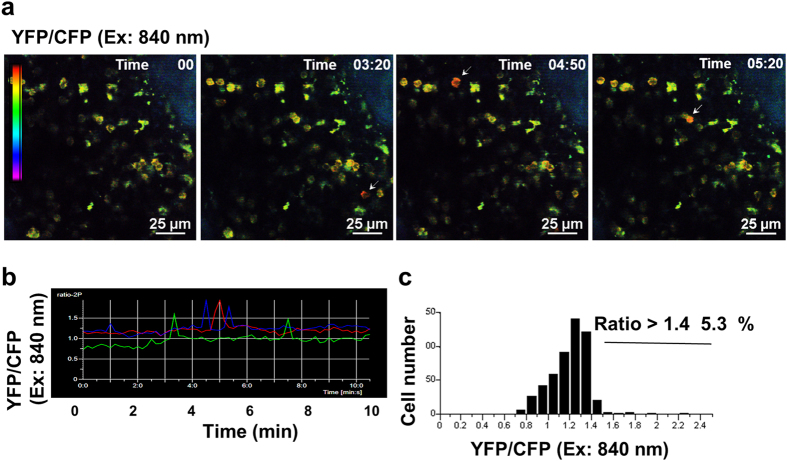
Representative Ca^2+^ signaling images in the bone marrow of a YC3.60^flox^/CD19-Cre mouse. (**a**) Representative Ca^2+^ signaling image in the bone marrow of a YC3.60^flox^/CD19-Cre mouse. Intravital imaging of the bone marrow was performed using two-photon microscopy. Only ratiometric images (YFP/CFP at excitation of 840 nm) are shown. Cells exhibiting FRET signals are indicated by arrows. Results are representative of three independent experiments (*n* = 3 mice). Scale bar, 25 μm. (**b**) Time course of intracellular Ca^2+^ fluxes. Ratiometric intensities (YFP/CFP at excitation of 840 nm) of indicated cells in (**a**) were measured for 10 min at 10 s intervals. (**c**) Distribution of time-integrated intracellular Ca^2+^ concentrations of B cells. *n* = 50, frame = 61.

We also analyzed the primary lymphoid organ (*i.e.*, bone marrow) where B cells originate. YC3.60 intensities were somewhat similar between pro-B cell to mature B-cell stages, although YC3.60^+^ frequencies were different (Supplementary Fig. S3b). YC3.60 intensities of plasma cells (B220^low/−^) are higher than those of mature B cells. 2P microscopy enables detection of fluorescent signals from the cells through bone tissue. Cell-to-cell contacts frequently occurred, and the Ca^2+^ concentration was transiently elevated (Fig. 4a,b, Supplementary Video S5). Approximately 5% of B cells in the bone marrow showed Ca^2+^ signals (Fig. 4c). Thus, we visualized the real-time dynamics and Ca^2+^ signals of B cells occurring in the bone marrow.

### Intravital imaging of Ca^2+^ signaling in the spleens of immunized YC3.60^flox^/CD19-Cre mice

**Figure 5:**
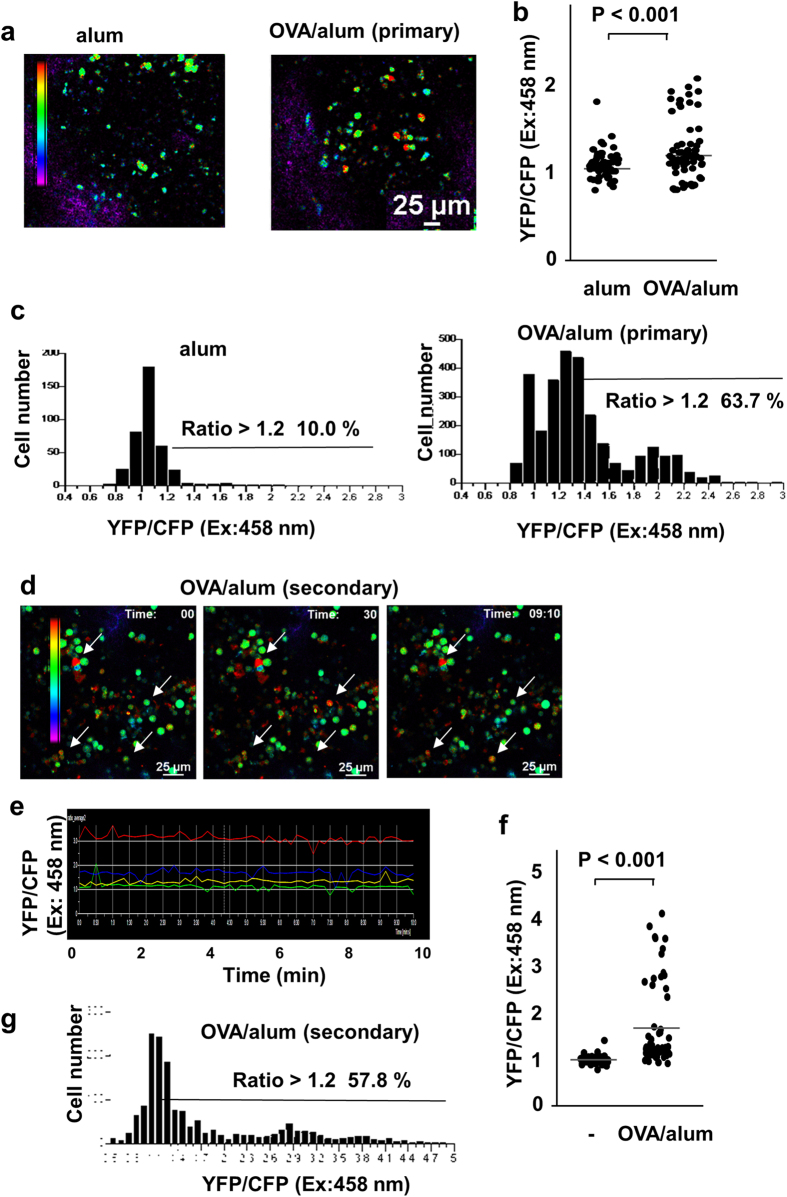
Representative Ca^2+^ signaling images in the spleen of an OVA/alum-immunized YC3.60^flox^/CD19-Cre mouse. (**a**) Images of Ca^2+^ signaling in the spleen of a non-immunized mouse and primary-immunized YC3.60^flox^/CD19-Cre mouse. YC3.60^flox^/CD19-Cre mice were immunized with OVA/alum; after 14 days, analysis of spleens of the mice by intravital imaging was performed using confocal microscopy. Images were obtained every 2 s. Representative Ca^2+^ images based on the ratio (YFP/CFP at excitation 458 nm) are shown (n = 3). (**b**) Ratiometric intensities (YFP/CFP at excitation 458 nm) of splenic B cells of control (alum only) and OVA-immunized mice. P < 0.01 (t-test). Bars denote mean values. *n* = 52. (**c**) Distribution of time-integrated intracellular Ca^2+^ concentrations of B cells of non-immunized and OVA-immunized mice. *n* = 30, frame = 13 (alum) and *n* = 16, frame = 90 (OVA/alum). Percentages of cells: ratios > 1.2 are indicated. Representative data of three mice are shown. (**d**) Images of Ca^2+^ signaling in the spleen of a secondary-immunized YC3.60^flox^/CD19-Cre mouse. YC3.60^flox^/CD19-Cre mice were immunized with OVA/alum and boosted on day 30; after 7 days, analysis of the spleens of the mice was conducted with intravital imaging. Representative Ca^2+^ images based on the ratio (YFP/CFP at excitation of 458 nm) at indicated time points are shown. Cells exhibiting FRET signals are indicated by arrows. Results are representative of at least three independent experiments (*n* > 3 mice). (**e**) Time course of intracellular Ca^2+^ fluxes. Ratiometric intensities (YFP/CFP at excitation of 458 nm) of indicated cells in (**a**) were measured every 5 s for 10 min. (f) Ratiometric intensities (YFP/CFP at excitation of 458 nm) of splenic B cells of non-immunized and OVA-immunized mice. P < 0.01 (t-test). Bars denote mean values. *n* = 50. (**g**) Distribution of time-integrated intracellular Ca^2+^ concentrations of B cells of non-immunized and OVA-immunized mice. *n* = 50, frame = 61. Percentages of cells: ratios > 1.2 are indicated.

To evaluate the effect of antigen-immunization on Ca^2+^ signals in B cells under physiological conditions, we conducted intravital imaging of the spleen of immunized YC3.60^flox^/CD19-Cre mice. We immunized YC3.60^flox^/CD19-Cre mice with OVA/alum and examined Ca^2+^ signaling in B cells. Fourteen days after primary immunization, many B cells exhibited constitutively high intracellular Ca^2+^ concentrations, although images were obtained with some intervals. In contrast, alum without OVA induced marginal Ca^2+^ responses (Fig. 5a–c). Furthermore, secondary immunization induced higher Ca^2+^ levels in B cells, although Ca^2+^ response was induced in similar frequencies (Fig. 5d–g, Supplementary Fig. S4). Almost no B cells of non-immunized mice showed constitutive high Ca^2+^ concentration (Supplementary Video S6). In these mouse spleen, GC cells and plasmablasts/plasma cells were increased (Supplementary Fig. S3a). In addition, some B cells showed temporal Ca^2+^ fluxes (Fig. 5d,e, Supplementary Video S7). After 14 days of boost, many B cells with constitutive high Ca^2+^ were still present in the spleen (Supplementary Fig. S4 a and b), suggesting that the immunization-mediated, activated state of B cells was maintained for a long period (*i.e.*, a period lasting longer than 14 days). These results indicate that immunization induced robust Ca^2+^ fluxes in a large number of B cells. Thus, intravital imaging revealed real-time events of B-cell activation induced by immunization.

### Intravital imaging of Ca^2+^ signaling in the spleens of autoimmune-prone and autoimmune YC3.60^flox^/CD19-Cre mice

**Figure 6:**
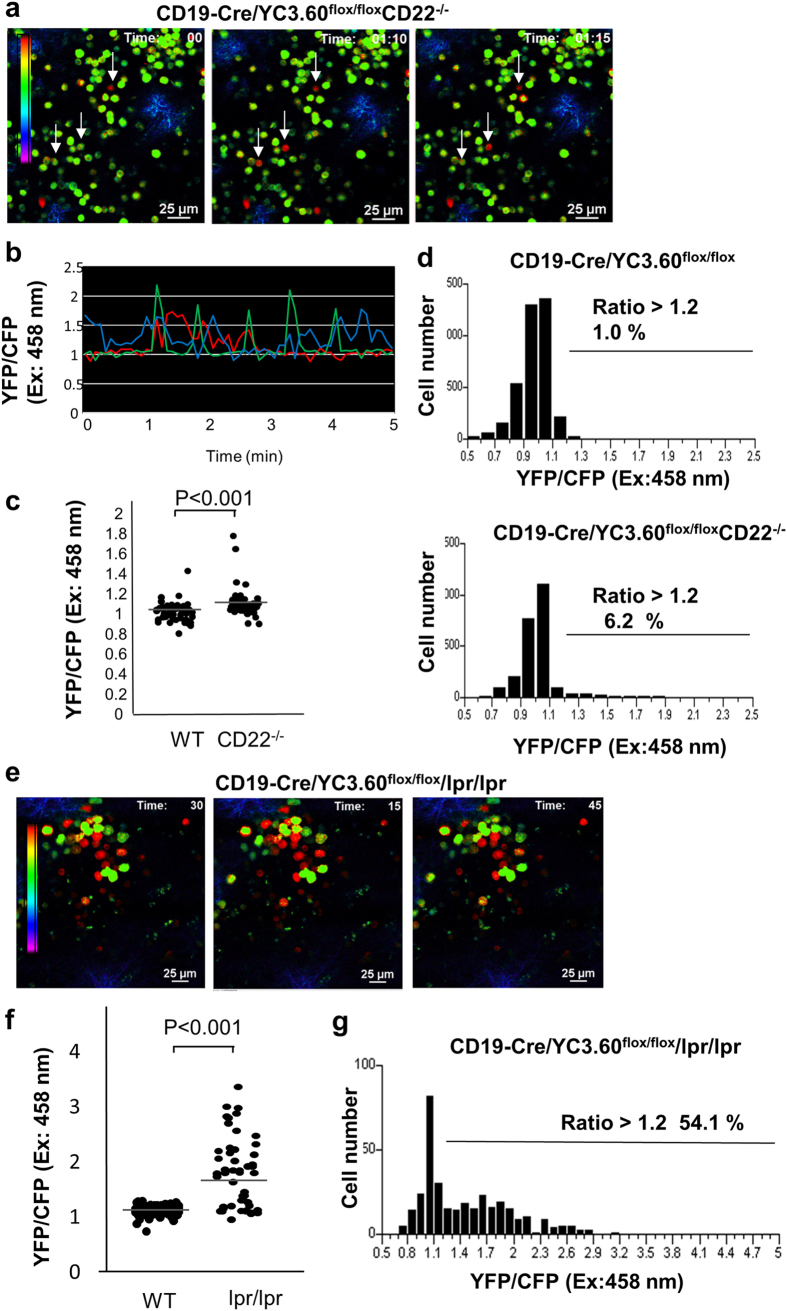
Representative Ca^2+^ signaling images in the spleen of autoimmune-prone YC3.60^flox^/CD19-Cre/CD22^−/−^ and YC3.60^flox^/CD19-Cre/lpr/lpr mice. (**a**) Images of Ca^2+^ signaling in the spleen of a YC3.60^flox^/CD19-Cre**/**CD22^−/−^ mouse. Representative Ca^2+^ images based on the ratio (YFP/CFP at excitation of 458 nm) at indicated time points are shown. Cells exhibiting FRET signals are indicated by arrows. Results are representative of at least three independent experiments (*n* > 3 mice). (**b**) Time courses of intracellular Ca^2+^ fluxes. Ratiometric intensities (YFP/CFP at excitation of 458 nm) of indicated cells in (**a**) were measured every 5 s for 5 min. (**c**) Ratiometric intensities (YFP/CFP on excitation at 458 nm) of splenic B cells from wild-type and CD22^−/−^ mice. Bars denote mean values. P < 0.01 (*t*-test). *n* = 50. (**d**) Distribution of time-integrated intracellular Ca^2+^ concentrations of B cells. Percentages of ratio > 1.2 were indicated. *n* = 50, frame = 61. (**e**,**f**) Representative Ca^2+^ signaling images in the spleens of YC3.60^flox^/CD19-Cre /lpr/lpr mice. (**e**) Images of Ca^2+^ signaling in the spleen of a YC3.60^flox^/CD19-Cre**/**lpr/lpr mouse. Representative Ca^2+^ images based on the ratio (YFP/CFP at excitation of 458 nm) at indicated time points are shown. (*n* = 3 mice). (**f**) Mean ratio (YFP/CFP at excitation of 458 nm) of splenic B cells from YC3.60^flox^/CD19-Cre and YC3.60^flox^/CD19-Cre**/**lpr/lpr mice. Bars denote mean values. P < 0.01 (*t*-test). *n* = 50. (**g**) Distribution of time-integrated intracellular Ca^2+^ concentrations of B cells. Bars denote mean values. *n* = 15, frame = 48.

To analyze the onset of autoimmune disease, we examined Ca^2+^ fluxes in B cells in autoimmune-prone mouse models, the CD22^−/−^ mouse and lymphoproliferation (lpr)/lpr mouse, both with C57BL/6 backgrounds. We crossed YC3.60^flox^/CD19-Cre mice with CD22^−/−^ mice, an autoimmune-prone mouse line lacking the inhibitory coreceptor CD22 of BCR^26^. Neither of the two mice showed any autoimmune traits, such as splenomegaly or expansion of germinal centers (Supplementary Fig. S2 and S3). GB B-cell population in the spleens of lpr/lpr mice was similar to that in wild-type mice (Supplementary Fig. S3a and d). CD22^−/−^ mice showed even fewer GC B cells than wild-type mice. Plasmablast/plasma-cell population was slightly increased in CD22^−/−^ and lpr/lpr mice. Intravital imaging of the spleen of YC3.60^flox^/CD19-Cre/CD22^−/−^ mice showed that a larger number of B cells exhibited Ca^2+^ signaling (Fig. 6a–d, Supplementary Video S8), although a lack of CD22 showed no severe defects in immune responses^27^,^28^,^29^,^30^. Furthermore, we examined B cells in another autoimmune-prone mouse model. Mice homozygous for the lpr mutation, which disrupts the expression of the Fas cell-surface molecule, develop severe lupus-like autoimmune disease, depending on their genetic backgrounds^31^,^32^. We analyzed the YC3.60^flox^/CD19-Cre/lpr/lpr mice on C57BL/6 in which a Fas^lpr^ mutation does not induce autoimmune disease (Supplementary Fig. S3). B cells with constitutively high Ca^2+^ were drastically increased in the spleen in these mice (Figs 6e–g), implying that they are B cells spontaneously activated by self-antigens. Furthermore, we examined YC3.60^flox^/CD4-Cre mice harboring the Fas^lpr^ mutation. Consistent with the results of B cells, Ca^2+^ fluxes were augmented in T cells of YC3.60^flox^/CD4-Cre/lpr/lpr mice (Supplementary Fig. S5), although the frequency was different. Thus, intravital imaging of YC3.60^flox^/lpr/lpr mice revealed abnormalities in intracellular Ca^2+^ flux in B and T cells. Taken together, analysis of intracellular Ca^2+^ signals in lymphocytes under physiological conditions exhibited abnormal traits in autoimmune-prone mice.

## Discussion

In this study, we successfully obtained conditional transgenic mice for visualization of Ca^2+^ signaling in specific cells. These mice enabled 5D intravital imaging in lymphoid tissues as well as in the bone marrow. Ca^2+^ flux frequency was increased in the CD22^−/−^ autoimmune-prone mouse, although there was no evidence of severe immunological disorder. Furthermore, in another autoimmune-prone model of C57BL/6lpr/lpr mice, Ca^2+^ fluxes in lymphocytes were found to be augmented. Taken together, the disorder of Ca^2+^ fluxes in lymphocytes appears to be a manifestation of autoimmune disease before the onset of pathological abnormality.

The conditional YC3.60 mice we generated expressed YC3.60 specifically on Cre expression. Furthermore, ubiquitous YC3.60 expression mice were also generated by crossing these mice with ubiquitous CAG promoter/enhancer-derived Cre mice^24^. This mouse line was first generated on May 31, 2012 and maintained for over 2 years without any distinct abnormality. To date, it has been reported that the transgenic expression of the Ca^2+^ biosensor in hosts induced problems, such as inactivation and disorders of the heart^16^. In contrast, the ubiquitous YC3.60 expression mice generated in our study showed no significant cardiac abnormalities. Although we do not know the exact reason for the generation of stable YC3.60 expression mice, we suspect that some effect of YC3.60 expression on biological functions is compensated for during maintenance of these mice over the generations.

As shown in the present study, we successfully detected Ca^2+^ signaling even in Peyer’s patches, which are altered by continual natural movements, such as intestinal peristalsis, heartbeat, and breathing. The ratiometric Ca^2+^ indicator YC3.60 enables monitoring of Ca^2+^ signaling in such conditions because motion-induced artifacts were corrected by its own internal control in the denominator of the ratio. As shown in Fig. 2b, fluorescence intensities of CFP and YFP were affected by motion. However, ratiometric indication compensated the influences of CFP and YFP and maintained the baseline. Thus, YC3.60 appears to be more adapted for *in vivo* imaging of tissues, such as the intestine, than are the single fluorescent protein-based Ca^2+^ sensors. Furthermore, because our mice adequately express YC3.60, YC3.60-expressing cells can be easily identified even in highly auto-fluorescent tissues, such as the intestine. Having access to YC3.60-expressing cells makes it possible to analyze both cellular dynamics and signaling in the cells of interest in living mice. Ca^2+^ signaling could be detected in the bone marrow, which is crucial in the development of hematopoietic cells and maintenance of immunological memory^33^. Furthermore, the YC3.60 gene has been stably inherited over 4 years, and its expression is restricted by Cre. Therefore, it is possible to trace the signature of the cells without losing expression of the transgene during long-term experiments, a feat that is not possible in virus vector-mediated gene transfer. Thus, YC3.60 transgenic mice are useful in intravital imaging of immune cells in various immunological tissues.

Ca^2+^ signaling was detected in the spleen, Peyer’s patches, and bone marrow in mice without any immunization protocol. These signals may be induced by environmental stimuli, such as commensal bacteria or environmental microbiota, even though procedures were conducted under SPF conditions. Therefore, these signals appear to be different from steady-state Ca^2+^ signals^34^. Furthermore, in the present study we showed sustained Ca^2+^ signals in B cells after immunization. However, previously oscillated Ca^2+^ signals induced by antigen-trapped dendritic cells have been reported^15^. This difference in Ca^2+^ signals may be associated with different experimental protocols, such as time period after immunization and quantity of antigen.

In the present study, we found that the Ca^2+^ flux frequency in autoimmune-prone CD22^−/−^ B cells was higher than that in wild-type B cells. CD22 is a B-cell specific membrane protein, which regulates BCR signaling as an inhibitory coreceptor^26^ and has been shown to be closely associated with autoimmune diseases^35^. However, CD22^−/−^ mice with a C57BL/6 background have been generated from four groups and none reportedly had immunological defects^27^,^28^,^29^,^30^, except for aged mice^28^. These results suggest that analysis of Ca^2+^ flux may warrant findings of the autoimmune-prone phenotype, although diagnostic phenotypes are absent. We also analyzed another autoimmune-prone model of Fas^lpr^ mutant mice with a C57BL/6 background. Fas-mediated apoptosis is crucial for lymphocyte selection to exclude self-reactive cells^36^,^37^. Although C57BL/6/lpr/lpr mice do not develop autoimmune disease^31^,^32^, we have demonstrated that C57BL/6/lpr/lpr mice (age, 6 months) do produce small amounts of autoantibodies^38^. Correlating well with this finding, the C57BL/6lpr/lpr mice exhibited a more salient phenotype in B-cell Ca^2+^ fluxes than that exhibited by CD22^−/−^ mice and had an abnormally high Ca^2+^ concentration similar to that observed in immunized mice. In these mice, plasmablasts/plasma cells were slightly increased. Ca^2+^ signals might be induced during these developmental process. Thus, intravital imaging of Ca^2+^ signals may improve earlier and precise detection of the onset of autoimmune diseases in the precritical stage, at least in mice. Although this system cannot be directly used in humans, detection of autoimmune diseases at the precritical stage may reduce suffering in patients, reduce clinical costs, and increase chance of cure.

A YC3.60 construct was inserted in chromosome 10 in conditional YC3.60 transgenic mice without interrupting any exons. This locus of insertion is located in a chromosome that is different from the ROSA26, which is utilized for developing various gene-targeted mice^39^,^40^. Therefore, it is possible to cross a panel of ROSA26-gene-targeted mice to analyze Ca^2+^ signaling in combination with other biological functions.

Under physiological conditions, our conditional YC3.60 mice are able to allow analysis of Ca^2+^ signaling in specific cells together with their dynamics. Furthermore, the ability to assess the progression of a disorder in real time is a valuable tool. Thus, analysis of the Ca^2+^ biosensor mice gives a deeper understanding of the mechanisms underlying physiological and pathological conditions.

## Methods

### Generation of YC3.60 reporter mice

A conditional YC3.60 expression construct was generated by inserting a loxP-flanked thymidine kinase promoter and neomycin phosphotransferase gene cassette as well as the YC3.60 gene downstream of the CAG promoter of the pCXN2 vector. The resulting plasmid vector was designated as pCAG-LoxPneoLoxP/Came-2. After *Bam*HI digestion, the linearized plasmid vector DNA was microinjected into the pronuclei of C57BL/6 fertilized mouse eggs and transferred to pseudopregnant females. The floxed YC3.60 reporter (YC3.60^flox^) mouse line was crossed with a CD19-Cre mouse line, which resulted in CD19^+^ cell-specific YC3.60 expression in YC3.60^flox^/CD19-Cre mice because of the loss of the loxP flanked neomycin cassette. The YC3.60^flox^ mouse line was crossed with CD4-Cre, Nestin-Cre, and CAG-Cre mouse lines. YC3.60^flox^/CD19-Cre mice were crossed with CD22^−/−^ and lpr mice to obtain YC3.60^flox^/CD19-Cre/CD22^−/−^ and YC3.60^flox^/CD19-Cre/lpr/lpr mice, respectively. All mice were maintained in our animal facility under SPF conditions in accordance with guidelines of the Institutional Animal Care and Use Committee of Tokyo Medical and Dental University. All experimental procedures on animals were approved by the Institutional Animal Care and Use Committee of Tokyo Medical and Dental University, and all experiments were carried out in accordance with approved guidelines.

### Flow cytometry

Ca^2+^ mobilization in YC3.60-expressing cells was analyzed by flow cytometry using CyAn ADP™ (Beckman Coulter) equipped with a 405-nm, solid-state laser as previously described. At 405-nm excitation, FRET intensity was calculated as the ratio of YFP to CFP intensity. Cell sorting was performed using a MoFlo XPD cell sorter (Beckman Coulter) in the Stem Cell Laboratory at our university. Antibodies with the following specificities were conjugated in-house: anti-CD19-Alexa647 and ant-B220-Alexa647. Streptavidin-PE and streptavidin-Alexa647 (which were obtained from BioLegend) were used as secondary reagents. Anti-CD3-PE, anti-CD4-PE, anti-GL-7-APC, anti-B220-Pacific Blue, anti-IgM-PE-Cy7 and anti-CD138-PE were also obtained from BioLegend.

### ELISA

An enzyme-linked immunosorbent assay (ELISA) was performed using the following antibodies: anti-IgM, anti-IgG, alkaline phosphatase-conjugated anti-IgM, and alkaline phosphatase-conjugated anti-IgG (Southern Biotech).

### Inverse PCR

Genomic DNA was prepared from a YC3.60^flox^ mouse. Then, DNA was digested with *Hpa*II and self-ligated. An inverse PCR test was performed using KOD FX Neo (TOYOBO) and a set of primers (5′-CGAGGGATCTTCATAAGAGAAGAGG-3′ and 5′-CCATAAGGTCATGTACTGGGCATAA-3′) and self-ligated genomic DNA as a template.

### Fluorescent microscopy

Organs and tissues were observed under a fluorescent stereoscope M165 FC with a luminous source FL600 (Leica).

### Intravital and *in vitro* microscope

Spleens or Peyer’s patches of anesthetized mice were imaged. Spleens or Peyer’s patches were surgically exteriorized, immobilized on a microscope stage, and maintained at 37 °C. A Nikon A1 laser scanning confocal microscope with a 20× objective and software NIS-Elements C was used for image acquisition. We used three dichronic mirrors (DM457/514 and DM405/488/561/640), and three bandpass emission filters (482/35, 540/30, 525/50, 595/50 and 700/75). YFP/CFP ratio was obtained by excitation at 458 nm. PE and Alexa-647 were excited at 488 nm and 633 nm, respectively. Images of purified cells in phosphate-buffered saline were also obtained as above. For 2P microscopy, we used a BX61WI/FV1000 upright microscope equipped with a ×25 water-immersion objective lens (XLPLN25XW-MP; Olympus, Tokyo, Japan), which were connected to a Mai Tai DeepSee HP Ti:sapphire Laser (Spectra Physics, Mountain View, CA). The excitation wavelength for CFP was 840 nm. We used an IR-cut filter BA685RIF-3, three dichroic mirrors (DM450, DM505, and DM570), and three emission filters [FF01-425/30 (Semrock) for the second harmonic generation image, BA460-500 (Olympus) for CFP, and BA520-560 (Olympus) for YFP]. Intravital microscopy of mouse calvaria bone tissues was performed using a protocol modified from a previous study; 10–14-week-old mice were anesthetized using isoflurane; the frontoparietal region of the skull bone was exposed, and the internal surfaces of bones adjacent to the bone marrow cavity were observed using multiphoton excitation microscopy. The imaging system was composed of a multiphoton microscope (A1-MP; Nikon) driven by a laser (Chameleon Vision II Ti: Sapphire; Coherent) tuned to 840 nm together with an upright microscope equipped with a 25× water immersion objective (APO, N.A. 1.1; Nikon). We used three dichronic mirrors (DM458, DM506, and DM561) and three bandpass emission filters (417/60, 480/40, and 534/30). Acquired images were analyzed with MetaMorph software (Universal Imaging, West Chester, PA) and Imaris Software (Bitplane AG, Zürich, Switzerland).

### Statistical Analysis

Statistical analysis was performed with unpaired Student’s t-test. A *P* value of <0.05 was considered statistically significant.

### Electrocardiography and ultrasound echocardiography

Experiments were performed on 8–12-week-old male mice. A surface ECG was recorded under anesthesia, where 0.5%–1.2% isoflurane was mixed with 100% oxygen. UCG was performed with the Vevo770 Imaging system using a 30 MHz probe (Visual Sonics, Toronto, Canada) under the same anesthesia. Left ventricular (LV) contractility and dimension were evaluated in short-axis view at the level of the papillary muscles.

## Additional Information

**How to cite this article**: Yoshikawa, S. *et al*. Intravital imaging of Ca^2+^ signals in lymphocytes of Ca^2+^ biosensor transgenic mice: indication of autoimmune diseases before the pathological onset. *Sci. Rep*. **6**, 18738; doi: 10.1038/srep18738 (2016).

## Supplementary Material

Supplementary Information

Supplementary Video S1

Supplementary Video S2

Supplementary Video S3

Supplementary Video S4

Supplementary Video S5

Supplementary Video S6

Supplementary Video S7

Supplementary Video S8
