# Supplementary material for: Intravital imaging of Ca2+ signals in lymphocytes of Ca2+ biosensor transgenic mice: indication of autoimmune diseases before the pathological onset
Source: Sci Rep. 2016 Jan 6;6:18738. doi: 10.1038/srep18738 (PMC4702216; doi:10.1038/srep18738)
Supplement: Supplementary Information [file srep18738-s1.pdf]

Supplementary information

Intravital imaging of Ca^{2+} signals in lymphocytes of Ca^{2+} biosensor transgenic mice: indication of autoimmune diseases before the pathological onset

Soichiro Yoshikawa[1], Takako Usami[2], Junichi Kikuta[3], Masaru Ishii[3], Tetsuo Sasano[4], Koji Sugiyama[5], Tetsushi Furukawa[5], Eiji Nakasho[6], Hiroshi Takayanagi[7], Thomas F. Tedder[8], Hajime Karasuyama[1], Atsushi Miyawaki[9], Takahiro Adachi[10]

[1]Department of Immune Regulation, Tokyo Medical and Dental University, Tokyo, 113-8519, Japan

[2]Laboratory of recombinant animals, Medical Research Institute, Tokyo Medical and Dental University, Tokyo, 113-8510, Japan

[3]Immunology and Cell Biology, Graduate School of Medicine, Osaka University, Suita 565-0871, Japan

[4]Department of Biofunctional Informatics, Tokyo Medical and Dental University, Tokyo 113-8519, Japan

[5]Department of Bio-informational Pharmacology, Medical Research Institute, Tokyo Medical and Dental University, Tokyo 113-8510, Japan

[6]R&D Group, Scientific Solutions Product Development Division, Olympus Corporation, Hachioji-shi, Tokyo 192-8507, Japan

[7]Department of Immunology, Graduate School of Medicine and Faculty of Medicine, The University of Tokyo, Tokyo 113-0033, Japan

[8]Department of Immunology, Duke University Medical Center, Durham, NC 27710

[9]Laboratory for Cell Function and Dynamics, Advanced Technology Development Group, Brain Science Institute, RIKEN, Wako, Saitama 351-0198, Japan.

[10]Department of Immunology, Medical Research Institute, Tokyo Medical and Dental University, Tokyo, 113-8510, Japan

Correspondence author: Takahiro Adachi,

Supplementary Figure S1. Generation and characterization of YC3.60^{flox}/CD19-Cre mice. (a) Schematic structure of the FRET-based C^{2+} biosensor YC3.60. CaM, calmodulin Ca^{2+} binding domain; M13, the CaM-binding domain of myosin light chain kinase. In the presence of Ca^{2+}, YC3.60 becomes compact by an interaction between CaM and M13, resulting in FRET between CFP (donor) to Venus (acceptor). (b) YC3.60 expression in B cells in YC3.60^{flox}/CD19-Cre mice. Splenic cells from two YC3.60^{flox}/CD19-Cre mouse lines were analyzed by flow cytometry. T and B cells were stained with an anti-CD3 mAb and an anti-B220 mAb, respectively. (c) B- and CD4^{+} T-cell populations in the spleen of YC3.60^{flox}/CD19$^{cre/+}$ mice. Splenic cells from YC3.60^{flox}/CD19-Cre mice, YC3.60^{flox} mice, and wild-type mice were stained for B220 and CD4. (d) YC3.60 expression in GC B cells and plasmablasts/plasma cells. GC B cells (GL-7) and plasmablasts/plasma cells (CD138) in spleen cells from OVA/alum-immunized YC3.60^{flox}/CD19-Cre mice after 14 days of immunization were stained. (e) Spleen weights of YC3.60^{flox}/CD19-Cre mice. $n = 3$. (f) Total cell, T cell and B cell numbers in the spleen of YC3.60^{flox}/CD19-Cre mice. $n = 3$. (g) Serum IgM and IgG antibody titers in non-immunized mice and OVA-specific IgG antibody titers in immunized mice. IgM (left), IgG (middle) and OVA-specific-IgG (right) antibody levels in sera from YC3.60^{flox}/CD19-Cre mice and wild-type mice were determined using ELISA ($n = 3$ each). (right) Sera were obtained from the mice before and after 14 days of OVA/alum immunization.

(e-g) Data are mean + SD. N.S: not significant (*t*-test). (h) Schematic structure of the conditional YC3.60 expression construct-integrated region in chromosome 10. DNA sequence obtained by inverse PCR was assigned to the region between *Spock2* and *Chsst3*. Positions of *Spock2* and *Chsst3* were derived from the NCBI Map Viewer (*Mus musculus*).

Supplementary Figure S2. YC3.60 expression in various Cre mice.

(a) YC3.60 expression in various Cre mice. Splenic cells from each mouse line were treated with anti-CD19 mAb and CD4 mAbs (top left). Splenic cells treated with isotype-matched control mAbs are shown (top right). Percentage of YC3.60^{+} cells in CD19^{+} cells (right) and CD4^{+} cells are shown (left). Percentages of YC3.60 positive cells in the total gated cells are indicated in histograms. Bright-field and fluorescent images of isolated spleen from each mouse line and the head of the indicated mouse lines are shown (right). (b) YC3.60 expression in a YC3.60^{flox}/nestin-Cre mouse. Only fluorescent images are shown.

Supplementary Figure S3. YC3.60 intensities in B cell populations in immunized mice and autoimmune-prone mice.

(a) YC3.60 intensities in B cell populations in the Peyer's patches and spleen. Cells from Peyer's patches and spleen of CD19-Cre/YC3.60^{flox} mice, spleen of OVA/alum-immunized CD19-Cre/YC3.60^{flox} mice, and spleens of CD19-Cre/YC3.60^{flox}CD22$^{-/-}$ and CD19-

Cre/YC3.60^{flox}/lpr/lpr mice were treated with anti-CD138 and GL-7 mAbs. Percentages of GC cells (GL-7^{+}) and plasmablasts/plasma cells (CD138^{+}) in the left panels were based on YC3.60^{+} cells (top). Middle and right panels were the results of total spleen cells. (b) YC3.60 intensities of B cell populations in bone marrow of CD19-Cre/YC3.60^{flox} mice. Bone marrow cells were stained with indicated mAbs. (c) Spleen weights of autoimmune-prone mice. n = 3. (d) Percentage of GC B cells in the spleen of autoimmune-prone mice. n = 3. GC B cells (GL-7^{+}) and total B cells (B220^{+}) in spleen were stained and analyzed by flowcytometry. (c and d) Wild-type, CD22$^{-/-}$ and lpr/lpr mice on the background of YC3.60^{flox}/CD19-Cre (eight week-old) were analyzed. Data are mean + SD. N.S: not significant (*t*-test).

Supplementary Figure S4. Representative Ca^{2+} signaling images in the spleen of an OVA/alum-immunized YC3.60^{flox}/CD19-Cre mouse.

(a) Images of Ca^{2+} signaling in the spleen of an OVA/alum-immunized YC3.60^{flox}/CD19-Cre mouse. OVA/alum were immunized to YC3.60^{flox}/CD19-Cre mice and, after 14 days of boost, the spleen of the mice were analyzed by intravital imaging. Representative Ca^{2+} images based on the ratio (YFP/CFP at excitation of 458 nm) at indicated time points are shown. Results are representative of three independent experiments (n = 3 mice). (d) Ratiometric intensities (YFP/CFP at excitation of 458 nm) of splenic B cells from control and immunized mice. Bars denote mean values. P: *t*-test. n = 50. (c, d) Three results based on the independent fields are

shown.

Supplementary Figure S5. Representative Ca^{2+} signaling images in the spleen of a YC3.60^{flox}/CD4-Cre/lpr/lpr mouse.

(a) Images of Ca^{2+} signaling in the spleen of a YC3.60^{flox}/CD4-Cre/lpr/lpr mouse. Representative Ca^{2+} images based on the ratio (YFP/CFP at excitation of 458 nm) at indicated time points are shown. Images were obtained using confocal microscopy. Cells exhibiting FRET signals are indicated by arrows. Results are representative of at least three independent experiments ($n > 3$ mice). (b) Ratiometric intensities (YFP/CFP at excitation of 458 nm) of splenic B cells from wild-type and lpr mice. Bars denote mean values. P = *t*-test. n = 50. (c) Distribution of time-integrated intracellular Ca^{2+} concentrations of B cells. Bars denote mean values. YC3.60^{flox}/CD4-Cre**,** $n = 40$, frame = 61; YC3.60^{flox}/CD4-Cre/lpr/lpr, $n = 40$, frame = 150.

Supplementary Video

Supplementary Video S1.

***In vitro* Ca^{2+} imaging of the spleen cells from a YC3.60^{flox}/CD19-Cre mouse.**

Ratiometric images (YFP/CFP at excitation of 458 nm) were measured every 2sonconds for 2 min (approximately 1 frame/second). Ionomycin (final concentration 5 μmol/L) was added at

the indicated time point. Real acquisition time is indicated (top). Results are representative of two experiments.

Supplementary Video S2.

Intravital Ca^{2+} imaging of B cells in the spleen of a YC3.60^{flox}/CD19-Cre mouse.

Ratiometric images (YFP/CFP at excitation of 458 nm) were measured for 40 seconds using confocal microscopy. Frame = 80. Cells exhibiting FRET signals in Fig. 2 are indicated. Real acquisition time is indicated (top).

Supplementary Video S3.

Intravital Ca^{2+} imaging of B cells in the Peyer's patch of a YC3.60^{flox}/CD19-Cre mouse.

Ratiometric images (YFP/CFP at excitation of 458 nm) were measured for 10 min using confocal microscopy. frame = 1129. Cells exhibiting FRET signals in Fig. 3 are indicated by arrows. Real acquisition time is indicated (top). Results are representative of 3 mice.

Supplementary Video S4.

3D Ca^{2+} images of the B cells in the Peyer's patch of a YC3.60^{flox}/CD19-Cre mouse.

Ratiometric images (YFP/CFP at excitation of 840 nm) are shown. Z-stack ratiometric images of 2-μm intervals up to a depth of 200 μm were obtained using 2P microscopy (Fig.3c and 3d).

3D structure image (510.00 x 473.00 μm. Depth, 200 μm).was generated by a Nis-Elements software.

Supplementary Video S5.

Intravital Ca^{2+} imaging of B cells in the bone marrow of a YC3.60^{flox}/CD19-Cre mouse.

Ratiometric images (YFP/CFP at excitation of 840 nm) are shown. Ratiometric images were measured for 10 min at 10-s intervals using 2P microscopy. Frame = 61. Cells exhibiting FRET signals in Fig. 3 are indicated by arrows. Real acquisition time is indicated (top). Results are representative of 3 mice.

Supplementary Video S6.

Intravital Ca^{2+} imaging of B cells in the spleen of a YC3.60^{flox}/CD19-Cre mouse.

The ratiometric images (YFP/CFP at excitation of 458 nm) are shown. Ratiometric images were measured every 2-s for 5 min using confocal microscopy. frame = 149. Results are representative of > 5 mice.

Supplementary Video S7.

Intravital Ca^{2+} imaging of B cells in the spleen of an OVA-immunized YC3.60^{flox}/CD19-Cre mouse.

Ratiometric images (YFP/CFP at excitation of 458 nm) are shown. Ratiometric images were measured for 10 min at 10 second intervals using confocal microscopy. frame = 61.Cells exhibiting FRET signals in Fig. 5 are indicated by arrows. Real acquisition time is indicated (top). Results are representative of 3 mice.

Supplementary Video S8.

Intravital Ca^{2+} imaging of B cells in the spleen of a YC3.60^{flox}/CD19-Cre CD22$^{-/-}$ mouse.

Ratiometric images (YFP/CFP at excitation of 458 nm) are shown. Ratiometric images were measured for 5 min at 5 second intervals using confocal microscopy. frame = 61. Cells exhibiting FRET signals in Fig. 6 are indicated by arrows. Real acquisition time is indicated (top). Results are representative of 3 mice.

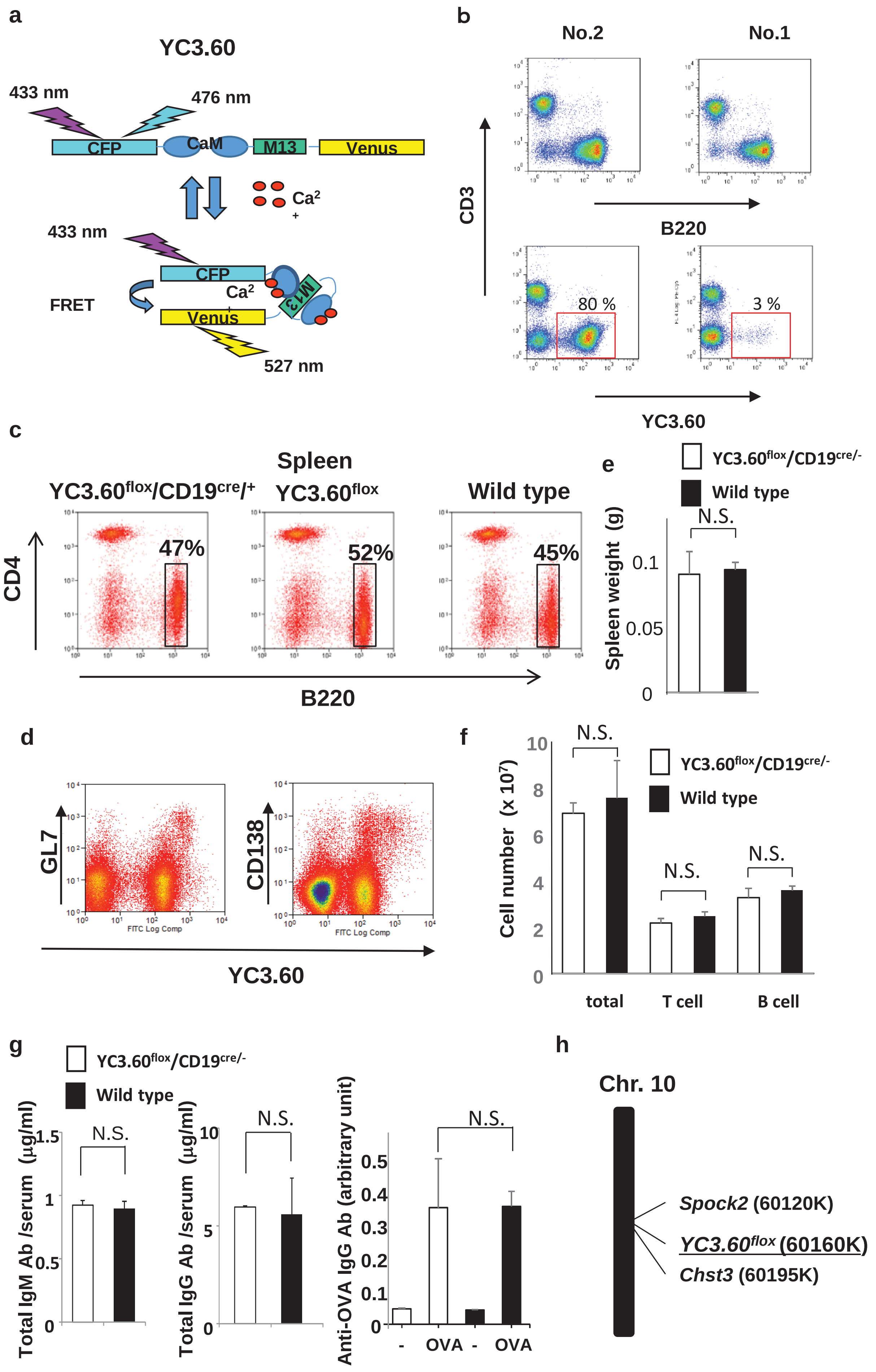

Supplementary Fig. S1

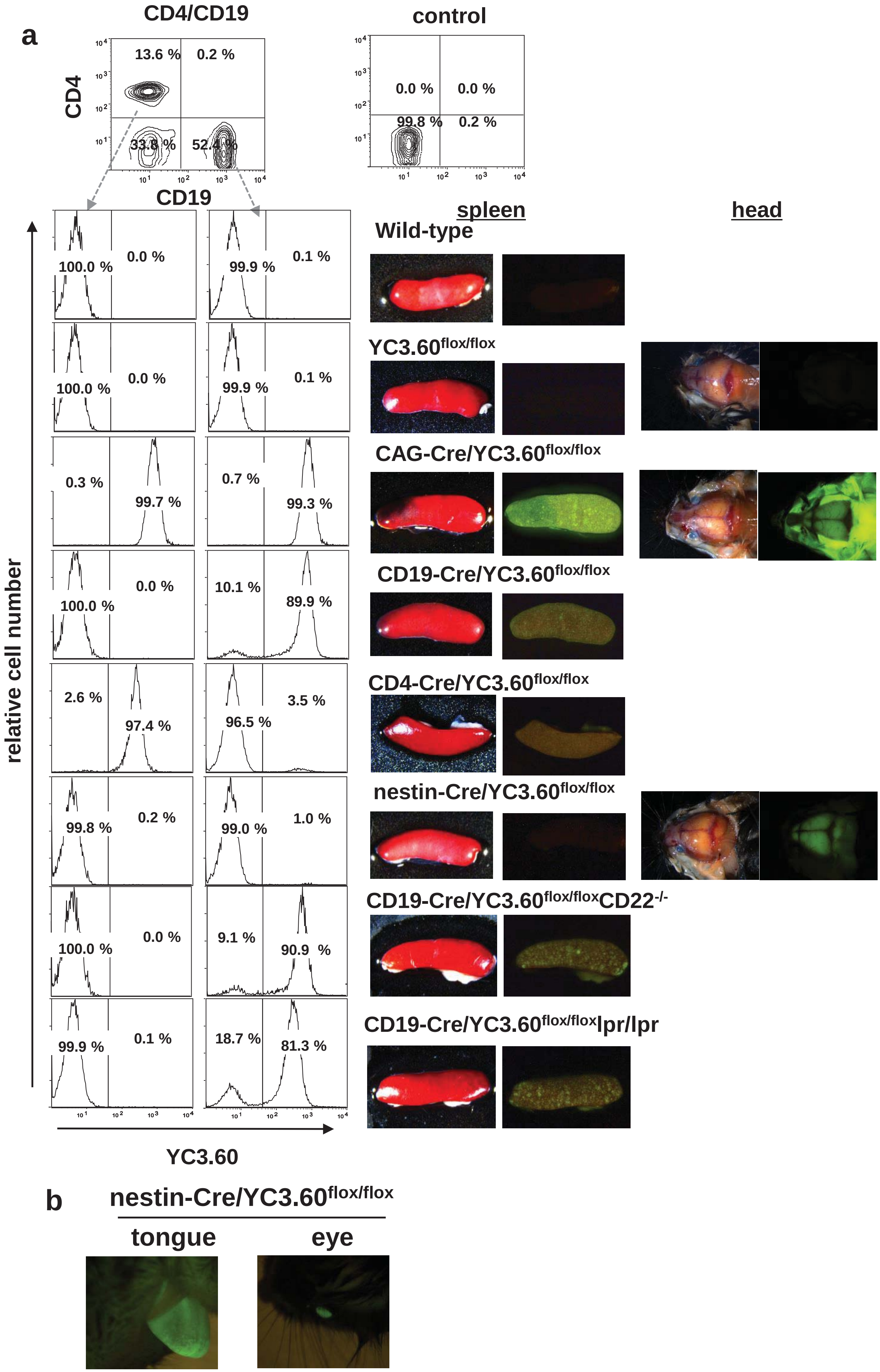

Supplementary Fig. S2

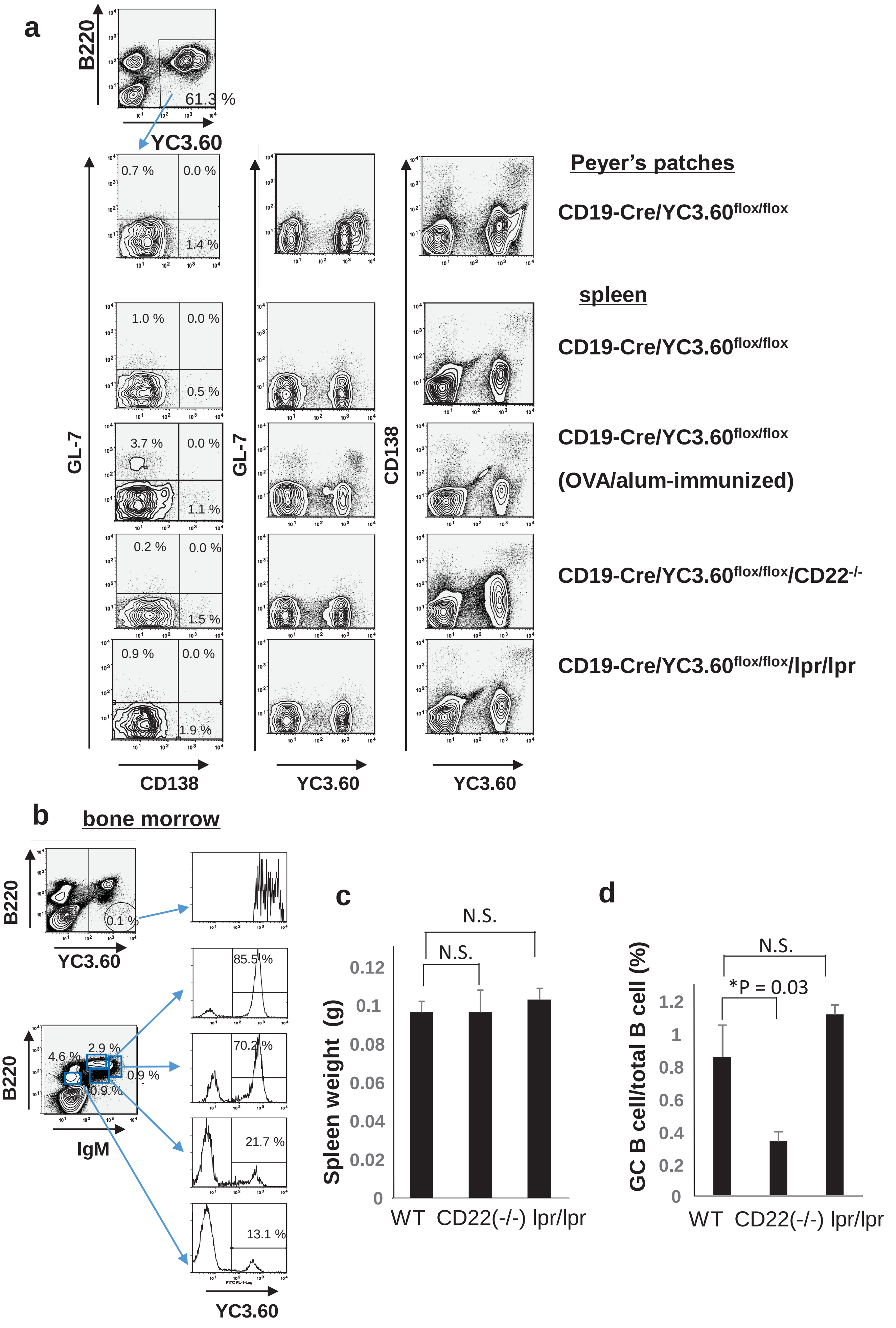

Supplementary Fig. S3

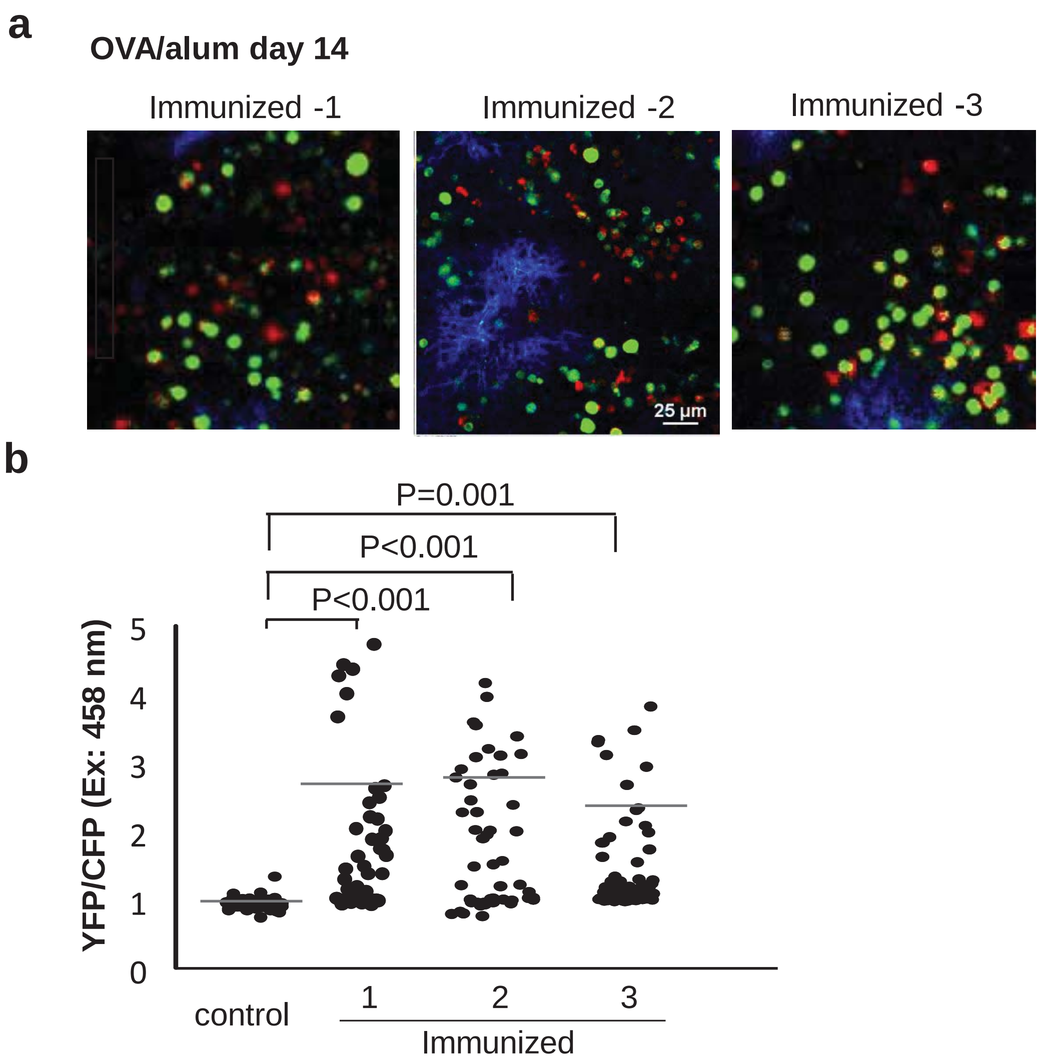
a
OVA/alum day 14
Immunized -1
Immunized -2
Immunized -3
25 μm
b
P=0.001
P<0.001
P<0.001
YFP/CFP (Ex: 458 nm)
5
4
3
2
1
0
control
1
2
3
Immunized

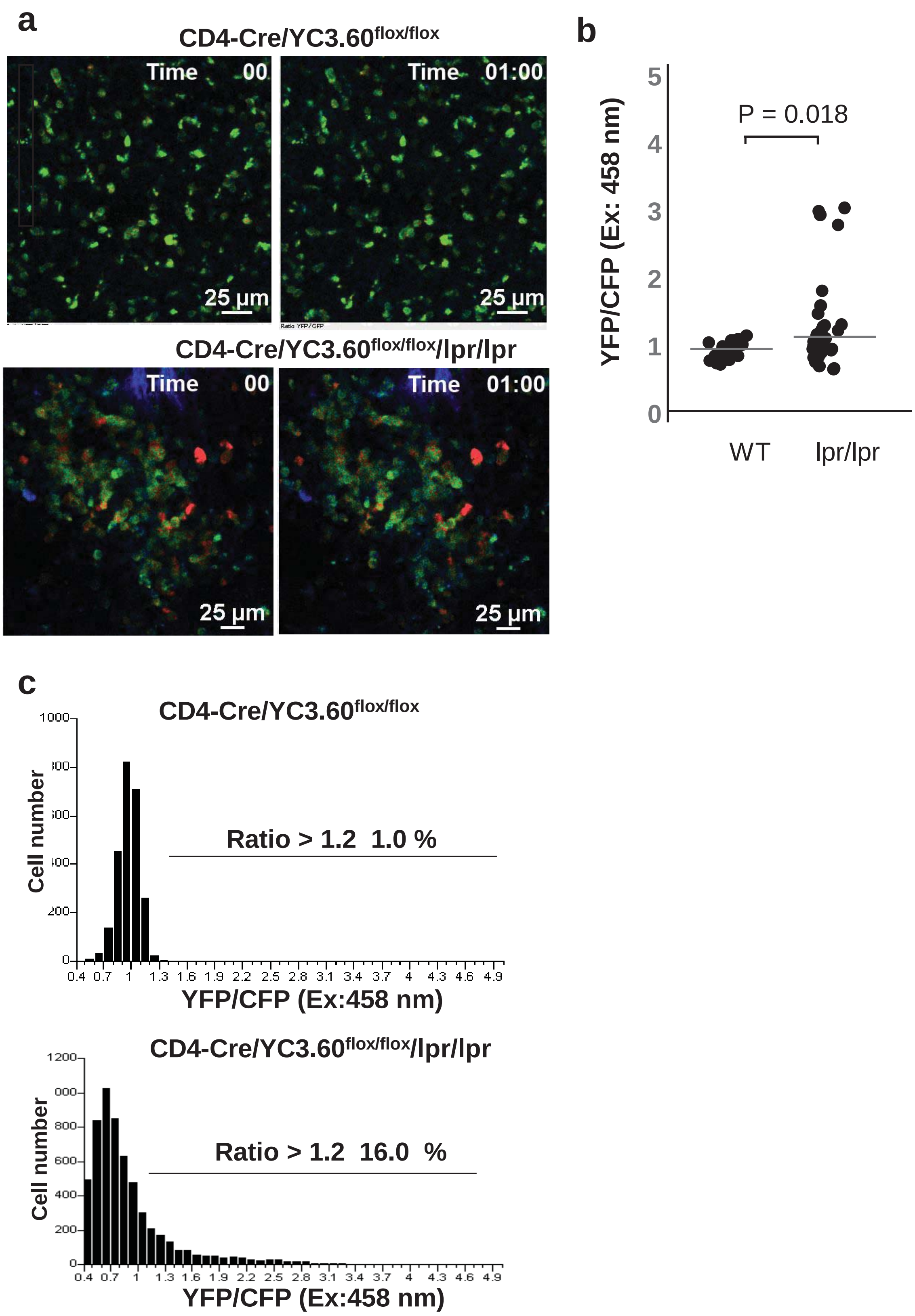

Supplementary Fig. S5
